# Role of Digital Health on Palliative Care: Umbrella Review

**DOI:** 10.2196/72104

**Published:** 2025-10-28

**Authors:** Junchen Guo, Yunyun Dai, Furong Chen, Chaoyi Liu, Sishan Jiang, Yonghong Hu, Yongyi Chen

**Affiliations:** 1 Department of Palliative Care Hunan Cancer Hospital Changsha China; 2 Australasian Health Outcomes Consortium University of Wollongong Wollongong Australia; 3 School of Nursing Guilin Medical University Guangxi China; 4 School of Nursing Guangzhou Medical University Guangzhou China

**Keywords:** digital health, telemedicine, telehealth, palliative care, systematic review, mobile phone

## Abstract

**Background:**

Digital health (DH) provides a valuable opportunity for accessible and efficient palliative care delivery. In recent years, an expanding body of systematic reviews and meta-analyses has examined DH-based interventions in palliative care. However, their conclusions regarding effects remain inconsistent, often constrained by methodological limitations and the variable quality of primary studies, making it difficult to form a coherent appraisal.

**Objective:**

This umbrella review aimed to examine, appraise, and synthesize previous systematic reviews and evaluate the role of DH-based services on palliative care, and to identify barriers to using DH-based services in these settings.

**Methods:**

Systematic reviews with or without meta-analysis focusing on DH within palliative care settings were considered eligible. Seven electronic databases, including PubMed, Web of Science, Embase, Cochrane Library, CNKI, Wangfang, and VIP, were searched for eligible studies published from inception to April 2024. The inclusion criteria were identified based on the principles of the PICOS (Population, Intervention, Comparison, Outcomes, and Study Type) framework. Two reviewers independently screened records and extracted data. Eligible studies were appraised for methodological quality using the JBI (Joanna Briggs Institute) Critical Appraisal Checklist for Systematic Reviews and Research Syntheses. A narrative synthesis, supported by tabulated summaries of the results, was used in this umbrella review.

**Results:**

A total of 25 systematic reviews (4 with meta-analyses) published between 2012 and 2024 met our inclusion criteria, most of which were evaluated as moderate quality. Reported outcomes ranged from symptom management effectiveness to psycho-social burden (ie, mood, distress, and emotional well-being), quality of life, caregiver burden, decision-making, cost-effectiveness, communication, self-efficacy and self-management efficacy, resource use, family empowerment, and acceptability. The effect of DH-based interventions for palliative care was basically consistent, with all included reviews reporting either significant improvements or noninferiority of DH-based interventions as compared to usual care. Technical challenges, organizational factors, ethical concerns, resource constraints, nonverbal communication, and perceptions were considered as barriers to the use of DH-based services.

**Conclusions:**

Across included reviews, DH was found to be beneficial or noninferior to standard care, with no reported adverse effects, supporting its safety and feasibility as a mode of service delivery. To ensure successful implementation and long-term sustainability, a multifaceted strategy is needed that integrates technological enhancements and training, organizational commitment, ethical safeguards, infrastructure development, and equitable access.

**Trial Registration:**

PROSPERO CRD42024539963; https://www.crd.york.ac.uk/PROSPERO/view/CRD42024539963

## Introduction

In recent years, there has been growing recognition of the importance of integrating palliative care early in the management of individuals with serious chronic illnesses. In 2017, the American Society of Clinical Oncology recommended the routine involvement of palliative care teams for patients with advanced cancer, highlighting the role of palliative care not only in symptom relief but also in supporting decision-making and improving quality of life [1]. While palliative care was initially developed with a primary focus on cancer, its role in noncancer chronic diseases is also increasingly acknowledged. With the rapid aging of the global population and the rising prevalence of conditions such as dementia and heart failure, the demand for palliative care has risen significantly compared to 30 years ago [2], and palliative care is now being integrated early in the treatment course for advanced diseases through embedded or stand-alone palliative care clinics [3]. However, attending an in-person palliative care clinic can still be challenging despite these recommendations. Due to disease progression and multiple organ failure, patients with advanced diseases often experience a significant decline in functional status, making it increasingly difficult for them to attend in-person clinic appointments [4,5]. Additionally, for those who live in remote or rural areas, accessing palliative care can be especially challenging due to limited medical resources; health care providers may only be able to provide services through intermittent telephone calls and infrequent home visits [6]. Providing accessible and efficient palliative care services throughout the disease trajectory from diagnosis to bereavement has become a major topic of concern.

With the rapid integration of global information and communication technology into health care systems, digital health (DH), a new health care service model, has gained significant attention in recent years. DH was defined as “an emerging field in the intersection of medical informatics, public health, and business, referring to health services and information delivered or enhanced through the internet and related technologies.” [7] In the Global Strategy on Digital Health 2020-2025, released by the World Health Organization in 2021, the benefits of DH are explicitly outlined. The strategy emphasizes that DH can enhance the efficiency and sustainability of health systems, facilitating the delivery of high-quality, affordable, and equitable care [8]. In the digital age, DH also provides a valuable opportunity in the field of palliative care, including facilitating better continuity of care, meeting the growing demands of palliative care services across remote areas with limited resources, and enhancing accessibility to high-quality palliative care delivery [9-11]. In addition to effectiveness, DH-based interventions can be a cost-effective option and may influence the use of palliative care services [12,13]. Given the increasing demand for palliative services due to an aging population and rising prevalence of chronic diseases, there is a compelling need to explore how DH technologies can be harnessed to enhance care delivery in this critical domain [7]. In recent years, an expanding body of research has examined DH-based interventions in palliative care, ranging from telemedicine consultations to remote symptom monitoring [11]. Despite this growth, the overall role of such interventions remains uncertain. Existing systematic reviews and meta-analyses have reached inconsistent conclusions, often constrained by methodological limitations and the variable quality of primary studies, making it difficult to form a coherent appraisal. For example, while Li et al [14] reported that DH-based palliative care improved quality of life in patients with advanced cancer, Yang et al [15] found no such benefit. Given the increasing number of systematic reviews assessing the effects of DH in palliative care, there is a need to consolidate these diverse findings into a single, high-level synthesis. An umbrella review, synthesizing findings across systematic reviews with or without meta-analyses through a standardized evaluative framework, is therefore warranted and represents one of the highest levels of evidence synthesis currently available [16]. This design enables direct comparison across existing syntheses, providing a broader perspective on common themes, methodological limitations, and evidence gaps.

Therefore, this umbrella review aimed to examine, appraise, and synthesize previous systematic reviews and evaluate the role of DH-based services on palliative care, and to identify barriers to using DH-based services in these settings. This synthesis will inform best practices, guide future research, and support policy makers in making evidence-based decisions to enhance palliative care services.

## Methods

### Study Design

An umbrella review was conducted in accordance with the methodology outlined in the JBI (Joanna Briggs Institute) Manual for Evidence Synthesis [16]. The protocol for this umbrella review was originally registered in PROSPERO (CRD42024539963). The PRIOR (Preferred Reporting Items for Overviews of Reviews) checklist used for reporting this umbrella review is provided in Multimedia Appendix 1.

### Eligibility Criteria

In this study, the inclusion criteria were identified based on the principles of PICOS (Population, Intervention, Comparison, Outcomes, and Study Type) framework [17], which was applied as follows. First, population: palliative care stakeholders regardless of age and ethnicity, such as patients (diagnosis of advanced cancer or a chronic disease unresponsive to active treatment), family caregivers, and health care professionals. Second, intervention: DH-based interventions provide information, support, and therapy (emotional, decisional, behavioral, and neurocognitive) for physical or mental health issues through technological or digital platforms (eg, website, videoconferencing, app, SMS text messaging, email, computer, and telephone) [18]. Third, comparison: no restrictions were set with regard to the types of comparators. Reviews were also considered eligible if they compared an eligible DH-based intervention arm to no intervention, including waitlist, usual care, or a sham intervention. Fourth, outcomes: changes in health or care process outcomes during DH-based palliative care; any outcomes about the efficacy or effectiveness dimension of evidence. Fifth, study type: systematic reviews published in English or Chinese, with or without meta-analysis. Scoping reviews were excluded as they do not typically assess study quality or synthesize intervention effects, making them less suitable for evaluating the strength of evidence in an umbrella review.

The exclusion criteria were as follows: (1) reviews focusing solely on outcomes for health care professionals and (2) papers reporting individual studies or protocols for forthcoming reviews, reviews of interventions that were not explicit and DH-based palliative care approaches, reviews with incomplete or irrelevant data, and unavailable reviews were also excluded in this study. Gray literature was excluded as the umbrella review focused solely on reviews of peer-reviewed, original studies [19].

### Search Strategy

The PubMed, Web of Science, Embase, Cochrane Library, CNKI, Wangfang, and VIP databases were used to systematically search for published studies from their inception to April 2024. Before refining the search terms, an initial search of PubMed for English studies and CNKI for Chinese studies was conducted to identify relevant search terms within the title and abstract. Subsequently, a search strategy combining free-text words and MeSH (Medical Subject Headings) terms was developed initially for PubMed and then adapted for each of the other databases. Building upon this foundation, the final search terms were tailored to the individual databases using Boolean phrases and operators; the key concepts of “palliative care” and “DH” were captured in each database. Moreover, a snowball method was also used to search the reference lists of the selected studies for reading and reference for a broader literature review. The detailed search terms, strategy, and results for each database were presented in Multimedia Appendix 2.

### Data Selection

The search results were first imported into EndNote software (EndNote V.X9.1; Clarivate). After removing duplicate records, one researcher (JG) uploaded the remaining citations from EndNote to the Covidence (Veritas Health Innovation Ltd) software. After that, 2 researchers (JG and FC) then independently screened the titles and abstracts within Covidence for relevance to the research questions. To be included, studies must meet the inclusion criteria. The remaining full-text papers were also independently assessed for eligibility by the same 2 researchers. Any disagreements at either the title or abstract or full-text screening stage were discussed and resolved through consensus or consultation with a third independent reviewer (YC).

### Data Extraction and Synthesis

Two researchers (JG and FC) extracted and entered the data independently using a predesigned standardized form in Excel (Microsoft Corp, 2022). Any discrepancies between the 2 researchers were resolved through discussion or the third reviewer. The following information from the included reviews were extracted and synthesized: general details of the reviews (ie, first author, publication year, aim, review design, country, years covered by review, the number of studies included in the review, participants, and type of DH intervention), categories of DH based services in palliative care, the effects of DH on palliative care related outcomes (ie, outcome measure and findings), and the quality appraisal methods used by review authors and the results for included studies.

Due to the heterogeneity of the included reviews, a narrative description accompanied by supportive tabulations of the results was adopted. Specifically, the role of DH will be assessed by categorizing the reported outcomes and tabulating data, indicating whether the effects of the intervention are positive, negative, or not statistically significant. To identify and classify barriers to the use of DH-based services, we applied an inductive content analysis following the methodological guidance of Elo and Kyngäs [20]. Content-specific terms and phrases extracted from the reviews served as the unit of analysis. Two reviewers independently coded the data, and categories were iteratively refined through discussion until consensus was reached.

### Assessment of Methodological Quality

The JBI Critical Appraisal Checklist for Systematic Reviews and Research Syntheses was used to assess the quality of each review paper [21]. The checklist includes 11 structured questions, and reviews were considered to be of high quality when all included studies achieved at least 50% of the maximum possible score [19]. The assessment was conducted independently by 2 researchers (JG and YD); any discrepancies between the 2 researchers were resolved through discussion or the third reviewer.

### Ethical Considerations

Ethical approval is not necessary for the umbrella review.

## Results

### Search Results

**Figure 1:**
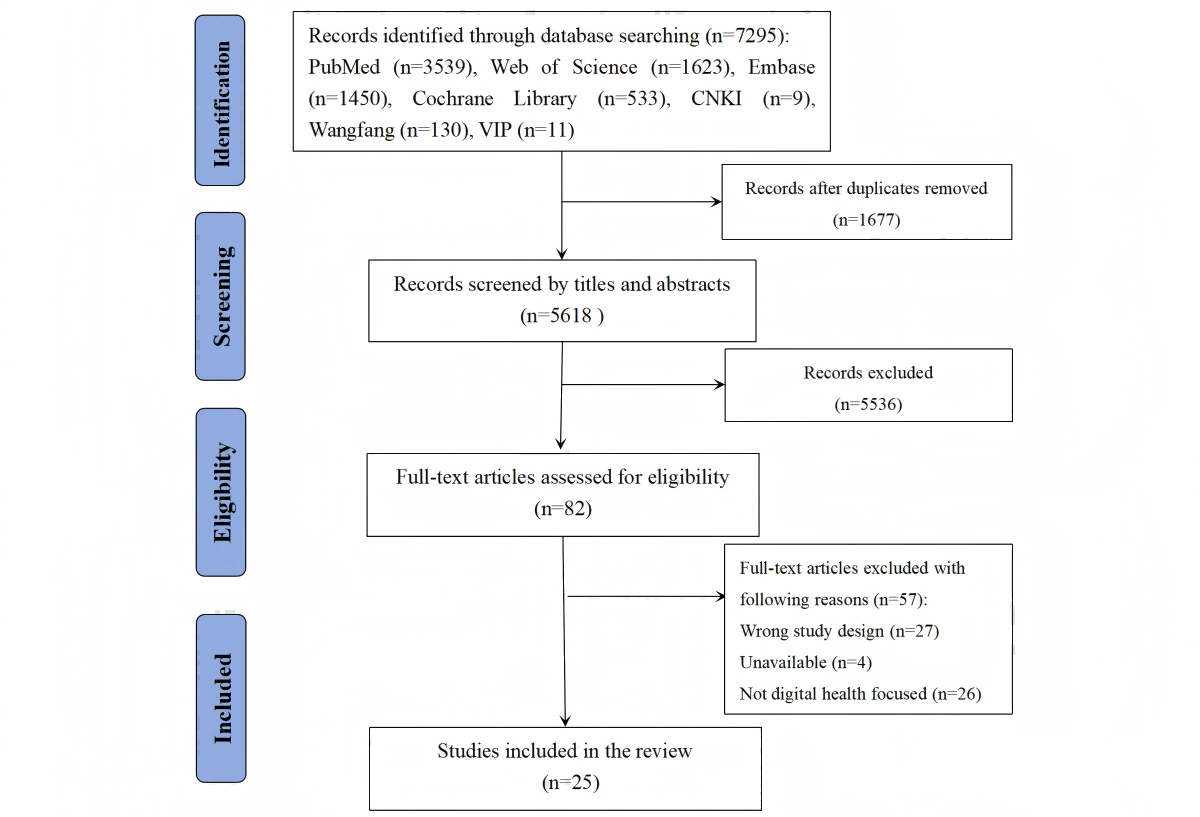
PRISMA flow diagram of study selection.

In this study, a total of 7295 records were retrieved from 7 electronic databases. After removing 1677 duplicates, 5618 records were screened by titles and abstracts, of which 5536 were excluded. The full texts of the remaining 82 records were retrieved for eligibility assessment, and after the full-text screening, 57 studies were excluded. Finally, 25 studies were included in this umbrella review. The PRISMA (Preferred Reporting Items for Systematic Reviews and Meta-Analyses) flow diagram of study selection is shown in Figure 1.

### Characteristics of Included Reviews

The included reviews were published between 2012 and 2024, with most of them having been conducted in United Kingdom (n=7) [22-28] and United States (n=7) [29-35], and the remaining 11 were conducted in China (n=4) [14,15,36,37], Australia (n=1) [38], Norway (n=1) [39], Chile (n=1) [40], France (n=1) [41], Greece (n=1) [13], Denmark (n=1) [42], and Colombia (n=1) [43]. The reviews included studies using various experimental designs. Three reviews consisted entirely of randomized controlled trials [14,15,36]. Additionally, 4 systematic reviews [14,15,25,29] also performed meta-analyses combining randomized controlled trial results on variables relevant to this umbrella review. Most reviews encompassed a range of study populations, including patients, family caregivers, and health care professionals. Two reviews [15,33] focused exclusively on DH-based interventions for caregivers, while 2 specifically addressed pediatric palliative care [26,38]. More details are shown in Table 1.

**Table 1:** Summary of included reviews.

Authors and year published	Aim	Review design	Country	Years covered by review	Included studies (N)	Participants (N)	Type of digital health intervention
Yang et al, 2024 [15]	To evaluate the impacts of telemedicine on the burden, anxiety, depression, and quality of life of informal caregivers of patients in palliative care.	Systematic review and meta-analysis	China	Database: inception to March 31, 2023	N=9 (2015-2023) all RCTs^a^	Informal caregivers of patients in palliative care (N=1215)	Websites Web conferences Applications Telephone
Dilhani et al, 2024 [22]	To summarize the range and characteristics of digital health interventions used to deliver palliative care and identify factors that influence their implementation and use.	Systematic review	United Kingdom	Database: inception to 4th May 2022	N=15 (2013-2022) Quantitative (n=5) Qualitative (n=3) Mixed methods (n=5) Quasi-experimental (n=1) Case series (n=1)	Patients with palliative care needs and their family caregivers (N=4857) Health care professionals engaged in palliative care (N=291)	ICTs^b^ Telephone consultation service Web Smartphone or mobile phone app Teleconsultation Palliative care information system
Johansson et al, 2024 [23]	To review the evidence for the clinical and cost-effectiveness of out-of-hours telephone advice lines for adults with palliative care needs living at home and their caregivers, and report service characteristics associated with effectiveness.	Systematic review	United Kingdom	Medline: 1946-February 9, 2023 Embase: 1974-February 9, 2023 CINAHL: 1937-February 9, 2023	N=21 (2000-2022) Quantitative (n=8) Qualitative (n=1) Mixed methods (n=5) Not specify the methodology (n=7)	Patients with palliative care needs and their caregivers (sample sizes were not reported)	Telephone advice lines
Chen et al, 2023 [36]	To describe the use of telepalliative care in patients with advanced disease and assess its effectiveness on quality of life, symptom burden, and other outcomes for patients and their caregivers.	Systematic review	China	Database: inception to November 27, 2021	N=30 (2000-2021) All RCTs	Patients with palliative care needs (N=19,665) and family caregivers (N=1153) including: Patients with advanced cancer Patients with end-stage heart failure Patients with advanced dementia Patients with end-stage renal disease Patients receiving ACPc	Videos Telephone Videoconferencing Websites Computers WeChat
Xu et al, 2023 [37]	To evaluate users’ reports of their satisfaction with telehealth palliative care during COVID-19, and to identify facilitators and barriers to implementation in palliative care during COVID-19.	Systematic review	China	From January 2020 to June 2022	N=18 (2020-2022) Quantitative (n=10) Qualitative (n=4) Mixed methods (n=4)	Patients with palliative care needs Family caregivers Palliative care providers (eg, clinicians and nurses) Telepalliative medicine volunteers	Telephone Video or videoconferencing Social media WhatsApp (WhatsApp LLC) Telecare platform
Sánchez-Cárdenas et al, 2023 [43]	To establish a telemedicine model of rural palliative care for patients with advanced cancer with difficulties accessing standard care.	Systematic review	Colombia	Between 2010 and 2021	N=14 (2006-2021) Observational: analytical (n=2) Observational: descriptive (n=4) Qualitative (n=2) Experimental-controlled clinical trials (n=6)	Patients with advanced disease candidates for palliative care (N=5485) Advanced cancer by staging disease severity (eg, stage III-IV) Estimated life time according to the patient’s functional life expectancy Modification of the therapeutic goal (patients without curative treatment or with palliative indications)	Applications Smartphones Smartwatches Telemedicine platforms Websites Email Telephone Videoconference
Steindal et al, 2023 [39]	To critically appraise and synthesize the findings from studies that investigated patients’ use of telehealth in home-based palliative care, focusing on the advantages and challenges experienced by patients.	Systematic review	Norway	From 2010 to 2022	N=41 (2011-2022) Quantitative (n=20) Qualitative (n=10) Mixed methods (n=11)	Patients with cancer and different life-limiting illnesses, including both cancer and noncancer diagnoses, motor neuron disease, heart failure, and chronic obstructive pulmonary disease (sample sizes ranged from 1 to 234 participants)	Video Web Webinar platform
Kamalumpundi et al, 2022 [29]	To critically evaluate the efficacy of web or mobile-based interventions impacting emotional symptoms in patients with advanced cancer.	Systematic review and meta-analysis	United States	From 1991 to 2019	N=23 (2011-2019) RCTs (n=17) Non-RCTs (n=6)	Patients with advanced cancer (N=2558)	Mobile app Web-based application
Goodman et al, 2021 [24]	To characterize the extent of engagement with telehealth interventions in patients with advanced, incurable cancer reported in the international literature.	Systematic review	United Kingdom	From database inception to December 31, 2020	N=40 (1996-2020) RCTs (n=16) Feasibility or pilot study (n=21) Observational study (n=3)	Patients with advanced and incurable cancer (N=4201)	Telephone sessions Tablet or computer kiosks Web-based messaging and communication Web conferences or videoconferencing Daily app Telehospice
Finucane et al, 2021 [25]	To capture, appraise, and synthesize the evidence represented in the systematic review literature on digital health interventions in palliative care.	Systematic meta-review	United Kingdom	From 2006 to 2020	N=21 (2007-2019) RCTs Non-RCTs Retrospective (including qualitative studies)	Patients, family members, caregivers with palliative care needs, and health professionals engaged in palliative care (sample sizes were not reported)	ICTs EHRsd Internet Weblogs Telephone Videoconference Simulators Social media Text messaging
Li et al, 2021 [14]	To explore the influence of telemedicine-based palliative care on the quality of life of patients with terminal cancer.	Systematic review and meta-analysis	China	From database inception to June 2020	N=8 (2015-2019) RCTs (n=8)	Patients with advanced and incurable cancer Intervention group (n=353) Control group (n=364)	Telephone conferences Telemedicine consultation system Website WeChat
Naoum et al, 2021 [13]	To identify and critically assess published studies on the economic evaluation of digital health interventions in the setting of palliative care.	Systematic review	Greece	From January 2010 to May 8, 2021	N=3 (2014-2021) RCTs (n=2) Observational study (n=1)	Patients with pediatric cancer (sample sizes were not provided) Adult patients with cancer (n=961)	Home telehealth program Video consultations Phone sessions eHealth self-management apps
Cameron and Munyan, 2021 [30]	To conduct a systematic review of the literature to discern available research and address the current state of the evidence related to telehospice services.	Systematic review	United States	From January 1, 2010, to May 1, 2020	N=13 (2010-2020) Qualitative studies (n=6) Quantitative studies (n=5) Mixed methods design (n=2)	Patients, caregivers with palliative care needs, and health professionals engaged in palliative care Sample sizes ranged from 1 to 15 in the qualitative studies and from 50 to 917 participants in the quantitative studies	Web-based videoconferencing Health care systems Phones and tablets Platform
Archer et al, 2021 [26]	To identify and synthesize the literature exploring the impact of all digital health interventions on the psychological outcomes of patients and families receiving pediatric palliative care.	Systematic review	United Kingdom	From database inception to July 27, 2020	N=3 (2012-2020) Observational study (n=2) Mixed methods design (n=1)	Patients, caregivers with palliative care needs (n=61)	Video Telephone Bespoke website Videoconferencing
Bienfait et al, 2020 [41]	To review the use of mHealth^e^ in the monitoring of patients with chronic pathologies, and to consider what could be adapted for palliative care patients at home.	Systematic review	France	From January 1, 2008, to June 18, 2018	N=22 (2008-2018) Literature reviews (n=8) Original papers (n=14)	Patients with chronic diseases (sample sizes were not reported)	Applications
Hancock et al, 2019 [27]	To describe the current use of telehealth in palliative care in the United Kingdom and evaluate telehealth initiatives against a digital service standard.	Systematic review	United Kingdom	From database inception to November 2017	N=30 (2010-2017) Qualitative studies (n=18) Mixed methods design (n=2) Protocols (n=3) Qualitative studies (n=7)	Patients, caregivers with palliative care needs (sample sizes ranged from 2 to 3594) Health professionals engaged in palliative care (sample sizes ranged from 1 to 105)	Telephone advice line Computer software Electronic patient records Videoconferencing CareHub (Canadian Virtual Hospice) device Smartphone apps
Jess et al, 2019 [42]	To review and synthesize current evidence regarding the use of video consultations in general and specialized palliative care to various patient groups.	Systematic review	Denmark	From database inception to January 15, 2018, and January 23, 2018	N=39 (2005-2017) Quantitative studies (n=10) Qualitative studies (n=10) Mixed methods design (n=14) Case studies (n=5)	Patients (n=2345), relatives (n=549) with palliative care needs, and health care professionals (n=252)	Videophones Computers with an integrated or external webcam Videoconference equipment Tablets
Allsop et al, 2018 [28]	To identify the development and use of mHealth in palliative care services in sub-Saharan Africa.	Systematic review	United Kingdom	From 1990 to April 2015	N=5 (2011-2014) Quantitative studies (n=1) Qualitative studies (n=4)	Patients, care givers with palliative care needs, and health care professionals engaged in palliative care (sample sizes are not provided)	Software Electronic health information systems Platforms
Bush et al, 2018 [31]	To assess whether the EHR effectively supports the multidisciplinary and complex nature of palliative care by examining its applications, methods, outcomes, and barriers to integration into clinical workflows.	Systematic review	United States	From 1999 to September 2017	N=30 (2008-2017) Quantitative studies (n=24) Qualitative studies (n=2) Mixed methods design (n=4)	Sample size ranged from 11 patients to 53,124 patients and 225 clinicians	EHR
Head et al, 2017 [32]	To explore published quantitative and qualitative research describing patient-reported outcomes of palliative telehealth intervention studies.	Systematic review	United States	From January 2006 to May 2016	N=11 (2006-2016) Quantitative studies (n=5) Qualitative studies (n=5) Mixed methods design (n=1)	Patients with palliative care needs (sample sizes ranged from 1 participant and 17 to 1352 participants)	Videophones Phones Patient self-monitoring equipment Computer program with internet access Smartphone apps
Zheng et al, 2016 [33]	To evaluate caregiver outcomes related to palliative telehealth interventions.	Systematic review	United States	From January 2003 to January 2015	N=9 (2007-2012) Quantitative studies (n=7) Qualitative studies (n=2)	The sample sizes of caregivers ranged from 8 to 217 participants	Videophones Phones Internet-based interventions Telehealth device
Ostherr et al, 2016 [34]	To identify the ICTs being used in end-of-life communication and compare the effectiveness of different ICTs in communication.	Systematic review	United States	From 1997 to 2013	N=38 (1997-2013) Quantitative studies (n=38)	End-of-life patients with palliative care needs (sample sizes ranged from 10 participants to 3112 participants)	Video Prototype website Telephone Videoconferencing Email prompt Telemonitoring Internet search Compact disc Fax PalmPilot (Palm Inc) SMS text messaging
Capurro et al, 2014 [40]	To systematically identify studies and analyze the effectiveness of eHealth interventions in palliative care and the information needs of people involved in the palliative care process.	Systematic review	Chile	From database inception to June 2012	N=17 (2004-2012) Quantitative studies (n=14) Qualitative studies (n=2) Case studies (n=1)	Patients or caregivers receiving palliative care (sample sizes ranged from 1 to 597 participants) Health professionals providing palliative care (sample sizes ranged from 7 to 160 participants)	Telephone Digital pen Process software
Bradford et al, 2013 [38]	To review the evidence for home-based telehealth in palliative care, particularly in pediatrics.	Systematic review	Australia	From database inception to February 22, 2012	N=33 (1998-2012) Review (n=4) Quantitative studies (n=11) Qualitative studies (n=18)	Patients or caregivers receiving palliative care (sample sizes ranged from 1 to 597 participants) Health professionals providing palliative care (sample sizes ranged from 7 to 160 participants)	Videoconferencing Videophone
Oliver et al, 2012 [35]	To systematically review the literature to discern available research and answer the question of the state of the evidence related to telehospice services.	Systematic review	United States	From 2000 to 2010	N=26 (2000-2010) Quantitative studies (n=19) Qualitative studies (n=7)	Patients or caregivers receiving palliative care, health professionals providing palliative care (sample sizes ranged from 2 to 3569)	Telephone advice lines Videophones Personal digital assistants Pen tablets Computers

^a^RCT: randomized controlled trial.

^b^ICT: information and communications technology.

^c^ACP: advance care planning.

^d^EHR: electronic health record.

^e^mHealth: mobile health.

### DH-Based Services in Palliative Care

Of the included reviews, the DH-based services in palliative care can be classified into the following 7 categories: communication and information exchange (asynchronous and synchronous), symptom management, psycho-social and spiritual support, advance care planning (ACP) and decision-making support, education and health literacy promotion, care coordination and reminders, and caregiver support. Among all these categories, DH-based communication and information exchange services were the most commonly used in palliative care, as reported in 21 reviews [13-15,22-30,32-38,40,42]. Additionally, 20 reviews [13-15,22,24-26,28-34,36,37,40-43] identified symptom management as the primary focus of DH-based interventions. Education and health literacy promotion were reported in 11 reviews [13,14,25,27-29,34-36,40,43], followed by care coordination and reminders in 9 reviews [14,24,27,29,31,32,38,41,43]. ACP and decision-making support were addressed in 7 reviews [14,15,25,31,34,36,37], psychosocial and spiritual support in 5 reviews [14,15,32,37,43], and caregiver support in 2 reviews [15,43].

### Effect of DH on Palliative Care–Related Outcomes

The summary statistics of the effect of DH on palliative care-related outcomes are shown in Multimedia Appendix 3 [13-15,23-34,36-38,40-42].

### Symptom Management

A total of 6 reviews [14,23,25,33,36,41] reported the effects of DH-based palliative care on patient symptom management outcomes. However, these reviews presented inconsistent findings. A total of 5 reviews [23,25,33,36,41] reported positive effects of DH-based palliative care on patient symptom management, 1 study [14] that conducted a meta-analysis showed DH-based palliative care had no statistically significant effect on improving the symptoms of advanced patients. There was a lack of evidence on the effects of DH-based palliative care on patient symptom management outcomes.

### Mood

A total of 8 reviews [14,15,29,32-34,36,38] reported the effects of DH-based palliative care on patient mood outcomes. Compared to usual care, some reviews reported that the DH-based palliative care can significantly reduce patients’ mood. However, 1 study [15] that conducted a meta-analysis showed DH-based palliative care had no statistically significant effect on reducing patients’ depression, but had a statistically significant effect on reducing anxiety. Additionally, 2 reviews [14,29] also conducted meta-analyses, reported that there was no difference between standard care and DH-based interventions in reducing the severity of anxiety and depression.

### Distress

A total of 2 reviews [29,34] reported the effects of DH-based palliative care on patient distress outcomes. However, these reviews also presented inconsistent findings. Reviews conducted by Kamalumpundi et al [29] indicated that DH-based palliative care interventions did not show a significant improvement in reducing distress severity compared with standard care. Another review conducted by Ostherr et al [34] found that the information and communications technologies can significantly decrease symptom distress in the intervention arm. There was a lack of evidence on the effects of DH-based palliative care on patient distress outcomes.

### Quality of Life

A total of 10 reviews [14,15,25,26,32-34,36,38,40] reported the effects of DH-based palliative care on patient quality of life. Most of the reviews showed there was no significant difference in quality of life between the intervention group with DH-based palliative care and the control group with standard care. However, the reviews also indicated that negative impacts were rarely observed, although there was no significant difference between the control group and intervention group.

### Psycho-Social or Emotional Well-Being

A total of 2 reviews [23,30] reported the effects of DH-based palliative care on patient psycho-social or emotional well-being. Both results of reviews reported that DH-based palliative care has a positive impact on patient psycho-social and emotional well-being.

### Caregiver Burden

A total of 3 reviews [15,33,36] reported the effects of DH-based palliative care on caregiver burden. The results of reviews reported that DH-based palliative care has a positive effect on caregiver burden.

### Decision-Making

A total of 3 [25,28,34] reviews reported the effects of DH-based palliative care on patient decision-making. However, the evidence was weak. One review [28] reported there was a statistically significant difference in developing ACP-related indicators between intervention groups and control groups. Other reviews [25,34] reported that the DH-based interventions potentially benefited ACP and could reduce decisional conflict.

### Communication

A total of 4 reviews [25,31,40,42] reported the effects of DH-based palliative care on communication. The results of all 4 reviews showed that DH-based palliative care can facilitate communication between patients and health care professionals.

### Self-Efficacy or Self-Management Efficacy

A total of 3 reviews [14,30,41] reported the effects of DH-based palliative care on patients’ or family caregivers’ self-efficacy or self-management efficacy. The results of all 3 reviews showed that DH-based palliative care can improve their self-efficacy or self-management efficacy.

### Family Empowerment

A total of 2 reviews [26,33] reported the effects of DH-based palliative care on family empowerment. The results of both reviews showed that DH-based palliative care can improve family empowerment.

### Resource Use

A total of 5 reviews [23,27,31,32,38] reported the effects of DH-based palliative care on hospital or intensive care unit (ICU) admission. Among them, 4 reviews [23,27,32,38] identified that patients who accessed DH-based interventions had a lower risk of hospital admission. One review [31] reported patients in the control group who received usual care had higher adjustment odds of ICU admission during the last 6 months and higher odds of death in the hospital or in the ICU. In addition to hospital or ICU admission, most reviews reported the positive effects of DH-based interventions in reducing emergency care use.

### Cost-Effectiveness

A total of 8 reviews [13,23,25,27,32,38,40,42] reported the effects of DH-based palliative care on costs. Most reviews [13,25,27,32,38,40] reported the positive effects of DH-based interventions on costs for patients, caregivers, or health care providers; several reviews identified that DH-based interventions can significantly cause a drop in hospital care costs [32,38].

### Acceptability (User Satisfaction and Patient and Care Giver Experiences) or Feasibility of DH

A total of 13 reviews [23,24,26-28,32,33,36-38,40-42] reported the acceptability or feasibility of DH. Most reviews [23,24,27,32,36,40-42] showed that the user experiences were overwhelmingly positive, such as the high levels of user satisfaction reported among patients and family members participating in DH-based interventions. Several reviews also found that DH was well-accepted among health care staff [27,41,42].

### Barriers to the Adoption of DH in Palliative Care Settings

**Figure 2:**
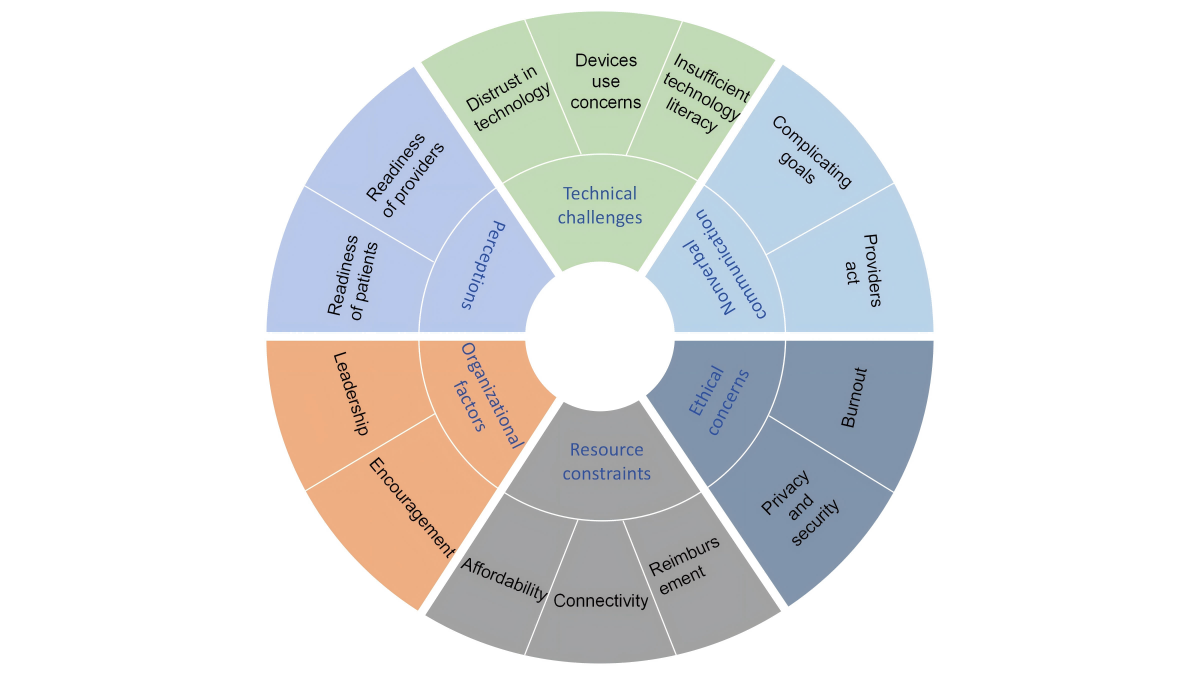
Barriers to the adoption of digital health in palliative care settings.

A total of 8 reviews [22,30,35,37-39,42,43] reported barriers to using DH in palliative care settings. Six main categories, including technical challenges, organizational factors, ethical concerns, resource constraints, nonverbal communication, perceptions, and 14 generic categories describing barriers to the use of DH-based services were identified in this review (Figure 2).

Technical challenges, including insufficient technology literacy and knowledge (eg, lack of professional training or being overwhelmed), device use concerns (eg, software malfunctions, confidentiality, and individualization), and distrust in technology, limited the use of DH-based services. The successful use of DH-based service was also influenced by organizational factors, including a lack of leadership and encouragement from the management. Additionally, ethical concerns are also a barrier to the use of DH-based services, including privacy and security, and burnout among health care professionals. Furthermore, the use of DH-based services was not supported in palliative care settings, due to a lack of nonverbal communication and perceptions, nonverbal communication such as professionals’ acting (eg, lacking in human touch or nonverbal cues) and complicating goals of care conversations, perceptions including the readiness of patients and professionals (eg, lack of experience or unfamiliarity). Other barriers, including resource constraints, such as affordability, connectivity (eg, internet access), and reimbursement challenges, also hinder the use of DH-based services.

### Methodological Quality

The quality appraisal methods used by the systematic review authors were presented in Multimedia Appendix 4 [13-15,22-43]. Most reviews used instruments from either the JBI or the Cochrane Collaboration, while 4 [23,24,37,39] used the Mixed Methods Appraisal Tool. Several authors also advised caution in interpreting results due to the variability in study quality. In this study, the results of critical appraisal of included systematic reviews were presented in Multimedia Appendix 5 [13-15,22-43]. The overall quality of the included reviews was moderate, and the high risk of bias was most often related to the critical appraisal, data extraction, and assessment of publication bias.

## Discussion

### Principal Findings

In 2016, a study [44] similar to ours investigated the use of telemedicine in palliative care. However, that study focused solely on the feasibility of telemedicine in this context and did not provide a detailed summary of the categories of DH-based services in palliative care, nor did it address the barriers and effects of DH applications in this field. To address the shortcomings of previous studies, this umbrella review systematically examined, appraised, and synthesized previous systematic reviews that specifically linked explicitly articulated DH-based interventions to palliative care services, and provided new, valuable insights for the continuous development and optimization of palliative care practices in the digital age.

In this review, we reported a total of 7 different DH-based services within palliative care settings, with the DH-based communication and information exchange services being the most widely used in this field. Communication and information exchange are an indispensable element in palliative care, and effective communication plays a crucial role in helping patients and their families understand the nature of the illness, the trajectory of the disease, and the available care options [45]. Clear, timely communication ensures that patients and their families are well-informed and can make educated decisions about the care they wish to receive [46]. Additionally, symptom management is also one of the crucial components of DH-based palliative care services. For most patients receiving palliative care, negative symptoms such as pain are commonly experienced. A longitudinal study funded by the National Institute on Aging identified that approximately 50% of these patients reported pain, and the rate tends to be higher among patients at home [47,48]. DH-based symptom management enables health care providers to track symptoms and vital signs from a distance, empowering patients and caregivers to record and manage symptoms proactively, contributing to a more comfortable experience [25,36]. Moreover, we also found that patient-centered education is one of the common services in DH-based palliative care. Education tailored to the specific needs of patients and their families not only enhances their understanding of the disease and care options but also fosters a sense of autonomy in managing symptoms and making decisions such as preferred place of death [49]. In the field of DH-based palliative care, digital platforms can serve as powerful tools for delivering personalized educational content, such as video tutorials, interactive modules, and real-time consultations with health care providers [25]. These resources can bridge the gap between clinical settings and home environments, ensuring continuity of care and reducing the burden on caregivers.

We synthesized evidence from 25 systematic reviews to evaluate 13 outcomes of DH-based interventions in palliative care. The overall role of DH-based interventions for palliative care was basically consistent, with all included studies reporting either significant improvements or noninferiority of DH-based interventions as compared to usual care. No reviews reported any adverse effects associated with DH-based interventions in palliative care settings. Some reviews reported that DH-based interventions were associated with significant reductions in caregiver burden [15,33,36], hospital or ICU admission [23,27,31,32,38], and costs [13,25,27,32,38,40], facilitates communication [25,31,40,42] and symptom management [23,25,33,36,41], improves self-efficacy [14,27,34] of patients and family caregivers, psycho-social or emotional well-being [23,30], and family empowerment [26,33]. For reduction in caregiver burden, a possible explanation for this result is that DH technologies can provide resources and tools for managing symptoms, educational materials, and establishing direct lines of communication between caregivers and health care professionals [50]. This support can alleviate the physical and emotional strain experienced by caregivers in managing the care of their loved ones, thereby improving their overall well-being. Additionally, DH technologies play a critical role in enabling the continuous monitoring of patient anthropometric and functional parameters [51]. These technologies, which include wearable devices, remote monitoring systems, and mobile health apps, allow health care providers to track vital signs, physical activity, and other health metrics in real-time. By enabling the early detection of health deterioration or complications, DH technologies facilitate timely interventions, potentially reducing the necessity for hospitalization or ICU admission [27]. A patient with advanced chronic heart failure may use a wearable device to monitor heart rate and fluid retention. Upon detection of any abnormalities, the health care team can intervene promptly, potentially preventing the need for an emergency hospital admission. This proactive approach not only improves patient care outcomes but also alleviates the strain on hospital resources and thereby cuts down on unnecessary health care expenses [31,32]. However, the review did not identify potential time savings associated with DH-based palliative care services due to a lack of sufficient evidence; future research could further explore the effects of this service on time costs. Moreover, DH tools such as continuing care service platforms often include educational resources and self-management features that empower patients and caregivers. By providing detailed information about the illness, treatment options, and symptom management strategies, these tools enhance the self-efficacy of both patients and their caregivers [14,41]. When individuals feel more knowledgeable and capable of managing their care, they may tend to experience better outcomes and higher satisfaction [14]. Furthermore, the results showed DH tools contribute to the psycho-social and emotional well-being of patients by offering accessible avenues for psychological support, including peer forums and tailored mental health resources. These features help alleviate feelings of anxiety, depression, and uncertainty [23,30]. Regular communication with health care providers and support networks through digital platforms further fosters a sense of connection and emotional safety, enhancing overall well-being [23,30]. At the same time, DH interventions promote family empowerment by facilitating shared decision-making and transparent communication. Features such as digital care planning and online family meetings enable family members to remain actively engaged in the care process, regardless of physical distance [14,30,41]. This collaborative model strengthens family cohesion and confidence in managing care needs, thereby enhancing the family’s sense of control, involvement, and preparedness throughout the palliative care journey. However, it needs to be acknowledged that current evidence on the effects of DH interventions on psycho-social and emotional well-being and family empowerment remains limited; more research is needed to confirm and expand upon these initial observations.

While identifying the potential benefits of DH-based palliative care services, this review also explored some of the barriers to using DH in palliative care settings. A frequently cited barrier in the included studies was technical challenges, including insufficient technology literacy and knowledge, and device use concerns, such as individualization. DH tools often require users to possess a certain level of familiarity with technology and digital interfaces. For health care providers, insufficient training in using DH tools can lead to suboptimal use, reduced confidence, and potential errors in patient care [22]. Similarly, patients and their families may struggle with the technical aspects of DH tools if they lack experience or support, which can hinder their ability to engage effectively with these technologies [37,39]. Additionally, DH technologies often need to be tailored to meet the specific needs and preferences of individual patients and their care plans. Most people who receive palliative care are older adults [52], and patients’ needs can be highly diverse and changeable; a one-size-fits-all approach may not be effective. Failure to adequately customize DH tools for individual patients may result in reduced efficacy and lower engagement, undermining the potential benefits of these technologies. Addressing these issues through targeted routine training and improved system reliability is essential for maximizing the role of DH technologies and ensuring their successful integration into palliative care practices. Moreover, resource constraints also hinder the use of DH-based services, particularly the lack of reliable internet access, a challenge that is especially prevalent in rural and remote areas due to limited infrastructure investment, lower population density, and the high costs associated with establishing and maintaining broadband networks in geographically isolated regions [22,43]. Future practitioners can collaborate with policy makers and technology providers to ensure affordable, reliable internet access in resource-limited communities. Emphasizing community engagement and scalable solutions can further help bridge the digital divide and enhance access to DH-based services in remote regions [53,54].

### Implications for Practice and Research

This umbrella review identifies several key barriers to the successful implementation and adoption of DH in palliative care, providing clear guidance for both clinical practice and future research (Textbox 1).

**Implications for practice and research.**

**Implications for practice**

- Develop continuous training programs for health care professionals, patients, and caregivers to improve digital literacy and confidence in using digital health (DH) tools.
- Encourage hospital and hospice leadership to actively endorse DH initiatives, allocate dedicated resources (eg, staff time or technical support), and embed digital solutions into routine clinical workflows to create an environment that supports adoption and long-term use.
- Implement robust policies and technical measures to protect patient privacy, data security, and reduce professional burnout.
- Expand internet infrastructure and establish reimbursement models to ensure equitable access to DH-based services, particularly in rural and underserved regions.

**Implications for research**

- Current evidence does not clearly demonstrate whether DH-based interventions save time for patients, caregivers, or providers. Future investigations, particularly randomized controlled trials and cost-effectiveness studies, are needed to clarify the impact of DH tools on time costs.
- Findings suggest potential benefits of DH in improving emotional support and family participation, but the evidence base remains limited. High-quality longitudinal and interventional studies are further needed to confirm these effects and explore mechanisms of action.
- Future research needs to assess the effectiveness of structured training programs, simplified user interfaces, and technical support models in improving adoption among patients, caregivers, and health care professionals with different levels of digital literacy.

### Limitations

One major limitation is the heterogeneity among the studies included in the review. The reviewed studies varied in terms of DH-based interventions assessed, study designs, patient populations, and outcome measures. This diversity may complicate the synthesis of results and limit the ability to draw broad, uniform conclusions. Therefore, our recommendations and conclusions should be considered with due caution. Additionally, DH technologies are rapidly evolving, and the pace of technological advancement means that the evidence reviewed may quickly become outdated. New technologies and innovations can emerge, altering the landscape of DH in palliative care and potentially affecting the relevance of earlier findings.

### Conclusions

This umbrella review synthesized evidence from existing systematic reviews to provide a comprehensive overview of the role of DH in palliative care. Among included reviews, DH was consistently reported to be either beneficial or noninferior compared with standard care, and no adverse effects were identified, indicating that DH is a safe and feasible mode of service delivery. To ensure successful implementation and long-term sustainability, a multifaceted strategy is needed that integrates technological enhancements and training, organizational commitment, ethical safeguards, infrastructure development, and equitable access. This work will support continued growth in the reach and effects of DH-based interventions to improve care outcomes for individuals receiving palliative care.

## Appendix

Multimedia Appendix 1 PRIOR checklist.

Multimedia Appendix 2 Search strategy.

Multimedia Appendix 3 Effect of DH on palliative care-related outcomes. DH: digital health.

Multimedia Appendix 4 Summary of quality appraisal of included studies in each systematic review.

Multimedia Appendix 5 Critical appraisal of included systematic reviews.

## Abbreviations

ACP: advance care planning
DH: digital health
ICU: intensive care unit
JBI: Joanna Briggs Institute
MeSH: Medical Subject Headings
PICOS: Population, Intervention, Comparison, Outcomes, and Study Type
PRISMA: Preferred Reporting Items for Systematic Reviews and Meta-Analyses

## Data Availability

The data used in this study are available from the corresponding author on reasonable request.
