# Supplementary material for: Role of Digital Health on Palliative Care: Umbrella Review
Source: J Med Internet Res. 2025 Oct 28;27:e72104. doi: 10.2196/72104 (PMC12605284; doi:10.2196/72104)
Supplement: Multimedia Appendix 2 [file jmir_v27i1e72104_app2.docx]

| **Database** | **Search strategy** | **Hits** |
| --- | --- | --- |
| PubMed | #1 "palliative care" [MeSH Terms] OR "terminal care" [MeSH Terms] OR "hospice care" [MeSH Terms] OR "hospices" [MeSH Terms] OR "palliative therapy" [MeSH Terms] OR "terminally ill" [MeSH Terms] OR "life support care " [MeSH Terms] OR "advance care planning " [MeSH Terms]  #2 "palliat*" [Title/Abstract] OR "palliative care" [Title/Abstract] OR "hospice care" [Title/Abstract] OR "hospices" [Title/Abstract] OR "terminal care" [Title/Abstract] OR "palliative therapy" [Title/Abstract] OR "terminally ill" [Title/Abstract] OR "end of life care" [Title/Abstract] OR "end-stage" [Title/Abstract] OR "life-limiting" [Title/Abstract] OR "life-threatening" [Title/Abstract] OR "life support care" [Title/Abstract] OR "advance care plan*" [Title/Abstract] OR "advance care planning" [Title/Abstract] OR "advance directives" [Title/Abstract] OR "advance disease" [Title/Abstract]  #3 #1 OR #2  #4 "digital health" [MeSH Terms] OR "telemedicine" [MeSH Terms] OR "[digital technology](https://www.ncbi.nlm.nih.gov/mesh/2051971)" [MeSH Terms] OR "Internet" [MeSH Terms]  #5 "telemedicin*" [Title/Abstract] OR "telemedical*" [Title/Abstract] OR "telemedicine" [Title/Abstract] OR "digital* health*" [Title/Abstract] OR "digital health" [Title/Abstract] OR "[digital technology](https://www.ncbi.nlm.nih.gov/mesh/2051971)" [Title/Abstract] OR "electronic* health*" [Title/Abstract] OR "telecommunications" [Title/Abstract] OR "telecare" [Title/Abstract] OR "telemonitor*" [Title/Abstract] OR "telemonitor" [Title/Abstract] OR "teleconsult" [Title/Abstract] OR "teleconference" [Title/Abstract] OR "video conferenc*" [Title/Abstract] OR "videoconferenc*" [Title/Abstract] OR "telephone*" [Title/Abstract] OR "telephone" [Title/Abstract] OR "telehealth" [Title/Abstract] OR "telehospice" [Title/Abstract] OR "remote" [Title/Abstract] OR "remote consult" [Title/Abstract] OR "ehealth*" [Title/Abstract] OR "ehealth" [Title/Abstract] OR "e-health*" [Title/Abstract] OR "e-health" [Title/Abstract] OR "mhealth*" [Title/Abstract] OR "m-health*" [Title/Abstract] OR "mhealth" [Title/Abstract] OR "m-health" [Title/Abstract] OR "mobile health" [Title/Abstract] OR "Internet" [Title/Abstract] OR "virtual" [Title/Abstract] OR "web" [Title/Abstract] OR "tablet*" [Title/Abstract] OR "smartphone" [Title/Abstract] OR "smart phone" [Title/Abstract] OR "app" [Title/Abstract]  #6 #4 OR #5  #7 "systematic review" [Title/Abstract] OR "systematic reviews" [Title/Abstract] OR "meta analysis" [Title/Abstract] OR "meta analyses" [Title/Abstract] OR "meta-analysis" [Title/Abstract] OR "meta-analyses" [Title/Abstract] | 3539 |
| Web of Science | #1 "palliative care" [Topic] OR "hospice care" [Topic] OR "hospices" [Topic] OR "terminal care" [Topic] OR "palliative therapy" [Topic] OR "terminally ill" [Topic] OR "end of life care" [Topic] OR "life support care" [Topic] OR "advance care planning " [Topic]  #2 "digital health" [Topic] OR "[digital technology](https://www.ncbi.nlm.nih.gov/mesh/2051971)" [Topic] OR "Internet" [Topic] OR "telehealth" [Topic] OR "telemedicine" [Topic] OR "ehealth" [Topic] OR "mhealth" [Topic]  #3 "systematic review" [Topic] OR "systematic reviews" [Topic] OR "meta analysis" [Topic] OR "meta analyses" [Topic] OR "meta-analysis" [Topic] OR "meta-analyses" [Topic]  #4 #1 AND #2 AND #3 | 1623 |
| Embase (via OVID) | #1 Palliative Care/  #2 ("palliative care" OR "hospices" OR "hospice care" OR "terminal care" OR "palliative therapy" OR "terminal care" OR "terminally ill" OR "end of life care" OR "life support care" OR "advance care planning ").ab,kw,ti.  #3 #1AND #2  #4 Digital Health /  #5("digital health" OR "digital technology" OR "Internet" OR "telehealth" OR "telemedicine" OR "ehealth" OR "ehealth").ab,kw,ti.  #6 #4 AND #5  #7 Systematic Review/  #8 ("systematic review" OR "systematic reviews" OR "meta analysis" OR "meta analyses" OR "meta-analysis" OR "meta-analyses").ab,kw,ti.  #9 #7AND #8  #10 #3 AND #6 AND #9 | 1450 |
| Cochrane Library | #1 "palliative care" [Title Abstract Keyword] OR "hospice care" [Title Abstract Keyword] OR "hospices" [Title Abstract Keyword] OR "terminal care" [Title Abstract Keyword] OR "palliative therapy" [Title Abstract Keyword] OR "terminally ill" [Title Abstract Keyword] OR "end of life care" [Title Abstract Keyword] OR "life support care" [Title Abstract Keyword] OR "advance care planning " [Title Abstract Keyword]  #2 "digital health" [Title Abstract Keyword] OR "[digital technology](https://www.ncbi.nlm.nih.gov/mesh/2051971)" [Title Abstract Keyword] OR "Internet" [Title Abstract Keyword] OR "telehealth" [Title Abstract Keyword] OR "telemedicine" [Title Abstract Keyword] OR "ehealth" [Title Abstract Keyword] OR "mhealth" [Title Abstract Keyword]  #3 "systematic review" [Title Abstract Keyword] OR "systematic reviews" [Title Abstract Keyword] OR "meta analysis" [Title Abstract Keyword] OR "meta analyses" [Title Abstract Keyword] OR "meta-analysis" [Title Abstract Keyword] OR "meta-analyses" [Title Abstract Keyword]  #4 #1 AND #2 AND #3 | 533 |
| Wanfang Database | #1 主题:("安宁疗护" OR "临终关怀" OR "姑息治疗" OR "姑息照护" OR "舒缓医疗" OR "生命末期" OR "终末期" OR "晚期")  #2 主题:("数字医疗" OR "远程医疗" OR "移动医疗" OR "移动健康" OR "远程照护" OR "远程服务" OR "数字健康" OR "智慧医疗" OR "互联网" OR "延续服务")  #3 主题:("系统评价" OR "meta分析" OR "综述")  #4 #1 AND #2 AND #3 | 130 |
| Chinese National Knowledge Infrastructure Database (CNKI) | #1 (SU=安宁疗护 OR SU=临终关怀 OR SU=姑息治疗 OR SU=姑息照护 OR SU=舒缓医疗 OR SU=生命末期 OR SU=终末期 OR SU=晚期)  #2 (SU=数字医疗 OR SU=远程医疗 OR SU=移动医疗 OR SU=移动健康 OR SU=远程照护OR SU=远程服务 OR SU=数字健康 OR SU=智慧医疗 OR SU=互联网 OR SU= 延续服务)  #3 (SU=系统评价 OR SU=meta分析 OR SU=综述)  #4 #1 AND #2 AND #3 | 9 |
| Weipu Database (VIP) | #1 M=("安宁疗护" + "临终关怀" + "姑息治疗" + "姑息照护" + "舒缓医疗" + "生命末期" + "终末期" + "晚期")  #2 M=("数字医疗" + "远程医疗" + "移动医疗" + "移动健康" + "远程照护" + "远程服务" + "数字健康" + "智慧医疗" + "互联网" + "延续服务")  #3 M=("系统评价" + "meta分析" + "综述")  #4 #1 AND #2 AND #3 | 11 |
