# Supplementary material for: Role of Digital Health on Palliative Care: Umbrella Review
Source: J Med Internet Res. 2025 Oct 28;27:e72104. doi: 10.2196/72104 (PMC12605284; doi:10.2196/72104)
Supplement: Multimedia Appendix 3 [file jmir_v27i1e72104_app3.docx]

**Table S1.** Effect of DH on palliative care related outcomes.

| **Outcome measure** | **Review** | **Findings** |
| --- | --- | --- |
| Symptom management | Johansson et al. 2024 [23] | The telephone advice lines provided valuable opportunity for patients and carers to ask about signs and symptoms. |
|  | Chen et al. 2023 [36] | 5/14 trials that reported patient symptoms showed statistically significant between tele-palliative care and control care groups with usual care. |
|  | Finucane et al. 2021 [25] | Some reviews described positive impacts of DHIs on symptom management, while most reviews identified inconsistent evidence or noted that evaluation of impact in many studies was lacking. |
|  | Li et al. 2021 [14] | The results of meta-analysis showed that DH-based palliative care had no statistically significant effect on improving the symptoms of patients with advanced cancer (SMD=0.10, 95%CI (-0.60, 0.80), *P*=0.78). |
|  | Bienfait et al. 2020 [41] | Most studies showed mHealth based palliative care can significantly improve symptom management in patients with chronic diseases. |
|  | Head et al. 2017 [32] | Among the 4 quantitative studies measuring patient symptoms, 2 studies found significantly lower levels of symptoms post intervention; another found no significant symptom improvement after the telehealth intervention. |
| Mood | Yang et al. 2024 [15] | The results of meta-analysis indicated that telemedicine has reduced the anxiety (SMD -0.49, −0.23, 95% CI −0.40 to -0.06; *P*=0.009; 95% PI -0.98 to 0.39) of informal caregivers, however, it did not affect depression (SMD -0.21, 95% CI -0.47 to 0.05; *P*=0.11; 95% PI -0.94 to 0.51). |
|  | Chen et al. 2023 [36] | 8/13 trials that reported patient mood showed statistically significant between tele-palliative care and control care groups with usual care. |
|  | Kamalumpundi et al. 2022 [29] | There was no difference between standard care and WMB interventions in reducing the severity of anxiety  (SMD −0.20; 95% CI−0.45 to 0.05, *I^2^*=72%; *P*=0.12) and the severity of depression (SMD −0.10; 95% CI−0.30 to 0.11; *I^2^*=73%; *P*=0.36). |
|  | Li et al. 2021 [14] | The results of meta-analysis showed that DH-based palliative care had no statistically significant effect on improving the mood of patients with advanced cancer [SMD=0.11, 95%CI (-0.35, 0.57), *P*=0.64]. |
|  | Head et al .2017 [32] | Patients in the treatment group had significantly lower anxiety, depression, and overall distress post intervention; another reported that anxiety improved. |
|  | Zheng et al. 2016 [33] | The caregiver anxiety score decreased after the intervention in two studies. |
|  | Ostherr et al. 2016 [34] | Most studies found that the ICTs can significantly reduce anxiety and depression, |
|  | Bradford et al. 2013 [38] | Of the five studies testing anxiety, one study found that families who received care by telehealth had a statistically significant reduction in parental anxiety. |
| Distress | Kamalumpundi et al. 2022 [29] | Compared with standard care, WMB interventions demonstrated no improvement in reducing the severity of distress (SMD−0.20; 95% CI−0.47 to 0.06; *I^2^*=60%; *P*=0.14). |
|  | Ostherr et al. 2016 [34] | Compared with standard care, studies found that the ICTs can significantly decrease symptom distress in intervention arm. |
| Quality of life | Yang et al. 2024 [15] | The results of meta-analysis indicated that telemedicine did not improve the QOL (SMD 0.35, 95% CI -0.20 to 0.89; *P*=0.21; 95% PI -2.15 to 2.85). |
|  | Chen et al. 2023 [36] | 10/15 included trials that reported patient QOL showed statistically significant between tele-palliative care and control care groups with usual care. |
|  | Finucane et al. 2021 [25] | Most reviews described improvements that were not statistically significant or positive impacts. Negative  impacts were rarely observed. |
|  | Li et al. 2021 [14] | The results of meta-analysis showed that DH-based palliative care can improve the quality of life of patients with advanced cancer [standardized mean difference (SMD) = 0.86, 95% CI (0.40, 1.32). |
|  | Archer et al. 2021 [26] | The study reported no differences in quality of life between caregivers in control and intervention groups over a 10-week period. |
|  | Head et al. 2017 [32] | Among the 2 studies measuring patient QOL, 1 study showed no significant difference between treatment  group and the control group. One of the qualitative studies concluded that telehealth had a positive effect on patient’s QOL. |
|  | Zheng et al. 2016 [33] | All the studies measuring caregiver QOL showed no significant difference after telehealth interventions. |
|  | Ostherr et al. 2016 [34] | Most studies found that the QOL in the intervention group (ICTs intervention) was significantly higher than that in the control group (usual care). |
|  | Capurro et al. 2014 [40] | Of the studies included, 3 studies reported that the eHealth interventions could improve the QOL. |
|  | Bradford et al. 2013 [38] | Of the five studies testing QOL, 4 studies showed there was no statistically significant differences were found in the QOL scores of the groups. |
| Psychosocial/ Emotional well-being | Johansson et al. 2024 [23] | Four studies identified that patients who accessed phonelines could relieve worry, and two studies indicated that could promote empowerment and independence. |
|  | Cameron and Munyan 2021 [30] | One study indicated that the telehospice could improve the caregivers’emotional comfort. |
| Caregiver burden | Yang et al. 2024 [15] | The results of meta-analysis indicated that telemedicine has reduced the caregiving burden (SMD -0.49, 95% CI-0.72 to-0.27; *P*<.001; 95% PI -0.86 to -0.13) of informal caregivers. |
|  | Chen et al. 2023 [36] | 3/4 trials that reported caregiver burden showed benefit in at least 1 domain at 1 or more time points. |
|  | Zheng et al. 2016 [33] | One study reported that telehealth based palliative care could significantly reduce caregiver burden. |
| Decision-making | Chen et al. 2023 [36] | 3/6 trials that reported ACP related indicators showed statistically significant between tele-palliative care and control care groups with usual care. |
|  | Finucane et al. 2021 [25] | The evidence from publications was weak, but points towards promising potential effects of EHRs for ACP. |
|  | Ostherr et al. 2016 [34] | One study indicated that the decisional conflict was improved in the intervention arm compared with control group. |
| Cost effectiveness | Johansson et al. 2024 [23] | One study identified that the advice lines incur slightly higher costs for OOH medicine prescription than other models of care, another study identified that the advice lines might generate future costsaving due to less urgent care use. |
|  | Finucane et al. 2021 [25] | Five reviews considered the financial implications of DHIs, with most reporting positive impacts of DHIs on costs for patients, caregivers or providers. |
|  | Hancock et al. 2019 [27] | Telehealth in palliative care can help reduce costs. |
|  | Jess et al. 2019 [42] | Four studies examined the economic implications  of video consultations in palliative care, and three out of four studies found that video consultations had economic advantages. |
|  | Head et al. 2017 [32] | Studies found that a telehealth intervention can significantly drop hospital care costs. |
|  | Naoum et al. 2021 [13] | The DH-based interventions incurred lower costs compared with usual care or no intervention and were considered cost saving and cost-effective. |
|  | Capurro et al. 2014 [40] | The eHealth intervention can significantly reduce costs. |
|  | Bradford et al. 2013 [38] | The home based telehealth intervention can significantly reduce hospital care costs. |
| Communication | Finucane et al. 2021 [25] | The role of DHIs to facilitate communication between patients, professionals and caregivers using phones, internet and computer systems. |
|  | Jess et al. 2019 [42] | Most studies found that video consultations can facilitate communication between patients and health care professionals. |
|  | Bush et al. 2018 [31] | Studies found that EHR can facilitate communication between patients and palliative care team. |
|  | Capurro et al. 2014 [40] | Of the studies included, 3 studies reported that the eHealth interventions could improve the communication. |
| Self-efficacy/ self management efficacy | Li et al. 2021 [14] | The results of meta-analysis showed that DH-based palliative care can improve self management efficacy in patients with advanced cancer (*P*＜0.05). |
|  | Cameron and Munyan 2021 [30] | One study showed significant improvements in caregivers’ skills and self-efficacy in supporting their children coping with their parents’ cancer. |
|  | Bienfait et al. 2020 [41] | Studies found that mHealth can improve self-efficacy in patients with chronic diseases. |
| Resource utilization (e.g: hospital/ICU admission, emergency care, primary care contacts and number of contacts needed from a DH provider) | Johansson et al. 2024 [23] | One study demonstrated that patients who accessed phonelines had lower risk of hospital admission in last week of life, and of dying in hospital. Two studies demonstrated that patients who accessed telephone advice lines were less likely to use emergency care services. |
|  | Chen et al. 2023 [36] | 3/7 trials that reported resource utilization showed statistically significant between tele-palliative care and control care groups with usual care. |
|  | Hancock et al. 2019 [27] | The use of telephone advice lines reported a reduction in admission. Of the 4 studies included, 2 studies showed there were less likely to use emergency care services. |
|  | Allsop et al. 2018 [28] | Text messaging can be used in resource-limited, oncological settings to potentially increase appointment adherence and improve rapport with patients. |
|  | Bush et al. 2018 [31] | Control group patients had higher adjustment odds of ICU admission during the last 6months, higher odds of death in the hospital or in the ICU. |
|  | Head et al. 2017 [32] | Studies found patients had fewer hospital admissions. Additionally, studies found patients showed lower utilization of clinical services, and avoided unnecessary hospitalization. |
|  | Capurro et al. 2014 [40] | Compared health care utilization before and after implementing the system, studies found that the number of emergency room visits by 19%, and the number of bed days by 77% after introducing the text messaging and videophone devices. |
|  | Bradford et al. 2013 [38] | Study reported a reduction in admissions to hospitals which was attributed to the use of these telehealth initiatives. |
| Family empowerment | Archer et al. 2021 [26] | The results from the MyQuality intervention showed a significant improvement in family empowerment over a 3-month period and increased feelings of control. |
|  | Zheng et al. 2016 [33] | The intervention group experienced significantly improved family functioning by reporting patients’ physical data through the telehealth device and accessing 24h telehealth support. |
| Acceptability (e.g:user satisfaction, patient and carer experiences) /feasibility/ usability of DH | Johansson et al. 2024 [23] | User experiences were overwhelmingly positive. |
|  | Chen et al. 2023 [36] | 5/6 trials found a significant improvement in tele-palliative care recipients’ satisfaction. |
|  | Xu et al. 2023 [37] | The satisfaction rates were high (66%-99%) among patients and family members who participated in telehealth consultations, but the satisfaction with family meetings was mixed. Compared with their clients, healthcare professionals were less likely to assess telehealth as satisfactory. |
|  | Goodman et al. 2021 [24] | Higher engagement occurred when a tablet, computer, or smartphone app was the mode of delivery. |
|  | Archer et al. 2021 [26] | One study reported an increased sense of identity and peace of mind in paediatric patients following two  sequential telehealth visits. |
|  | Bienfait et al. 2020 [41] | mHealth was validated acceptable compliance and acceptable staff response time threshold for generated alerts. |
|  | Hancock et al. 2019 [27] | Staff gave positive feedback- easy to use and increased job satisfaction. |
|  | Jess et al. 2019 [42] | A total of 18 studies reported on users’ perceptions of video consultations, and the majority of these studies concluded that patients, relatives, and health care professionals were positive toward the technology and its usage in palliative care. |
|  | Allsop et al. 2018 [28] | mHealth interventions can increase to the geographical coverage for accessing patients, and make appointment booking and reminders easier to perform. |
|  | Head et al. 2017 [32] | Four of the 11 studies reported that patients were satisfied after the telehealth intervention. |
|  | Zheng et al. 2016 [33] | Of the nine studies, four concluded that the telehealth intervention was feasible, five found overall caregivers’ satisfaction with the intervention. |
|  | Capurro et al. 2014 [40] | A high level of user satisfaction was reported in included studies. |
|  | Bradford et al. 2013 [38] | 6/18 papers presented case study examples that found that telehealth applications were well received by patients and clinicians. |

Abbreviations:SMD, standardized mean differences; QOL, quality of life; WMB,web or mobile-based; ICTs, Information and Communications Technologies; EHRs, Electronic Health Records; ACP, advance care planning; DHIs, digital health interventions.
