# Supplementary material for: Role of Digital Health on Palliative Care: Umbrella Review
Source: J Med Internet Res. 2025 Oct 28;27:e72104. doi: 10.2196/72104 (PMC12605284; doi:10.2196/72104)
Supplement: Multimedia Appendix 4 [file jmir_v27i1e72104_app4.docx]

| Authors and year published | Review design | Country | Quality appraisal of included studies – method | Quality appraisal of included studies – results |
| --- | --- | --- | --- | --- |
| Yang et al. 2024 [15] | Systematic review and meta-  analysis. | China | The methodological quality of the included studies using the Cochrane Collaboration’s tool for assessing the risk of bias | - 2 studies were determined as having a low risk of implementation bias. - Approximately half (4/9, 45%) the studies blinded the outcome assessors, and their risk of measurement bias was classified as low. - 3 studies were determined to have a high risk of attrition bias. - No selective reporting bias was detected in the included studies. - 4 studies were categorized as having a high risk of other biases |
| Dilhani et al. 2024 [22] | Systematic review | UK | Included studies were assessed for methodological quality using the relevant standard JBI critical appraisal instruments | Most quantitative studies and the quantitative component of the mixed-method studies  were of good quality |
| Johansson et al. 2024 [23] | Systematic review | UK | The quality of included studies was assessed using the Mixed Methods Appraisal Tool | - 7 studies could not be appraised as they lacked an explicit study aim or research question. - 14 studies were appraised and most had moderate or low scores ( one study scored 1 point; 6 studies scored 2 points; 4 studies scored 3 points; 2 studies scored 4 points; one study scored 5 points) |
| Chen et al. 2023 [36] | Systematic review | China | The Cochrane Collaboration's tool was used to assess the risk of bias of the literature. | Overall Risk of Biason:14 studies were assessed low; 9 studies were assessed moderate; 7 studies were assessed unclear. |
| Xu et al. 2023 [37] | Systematic review | China | The quality of the articles included was assessed using the Mixed Methods Appraisal Tool | - one study scored 2 points - 4 studies scored 3 points - 4 studies scored 4 points - 9 studies scored 5 points |
| Sánchez-Cárdenas et al. 2023 [43] | Systematic review | Colombia | The quality of the selected articles was assessed using the Newcastle-Ottawa Scale (observational studies, randomized clinical trials, and cross sectional studies);  Qualitative studies was assessed following the Standards for Reporting Qualitative Research. | 14 articles were classified as high-quality and were subsequently included in the review. |
| Steindal et al. 2023 [39] | Systematic review | Norway | The methodological quality of the included studies was independently appraised by the same pairs of authors using the Mixed Methods Appraisal Tool. | Most of the included qualitative studies (9/10, 90%) fulfilled all the quality criteria. 19 studies used a quantitative method; however, only 16% (n=3) of these studies used a randomized controlled trial design. Several of the mixed methods or multimethods studies did not sufficiently integrate or describe the integration of the quantitative and the qualitative components. |
| Kamalumpundi et al. 2022 [29] | Systematic review and meta analysis | USA | Methodological quality of RCTs and non-randomized studies were evaluated using the revised Cochrane Riskof-Bias (RoB) tool for randomized trials (RoB-2) and the Risk of Bias in Non-randomized Studies–of Interventions tool (ROBINS-I), respectively. | - Among the 6 non-RCTs, only one was determined to be at a high RoB, 5 studies were determined to be at moderate RoB, mostly due to a moderate RoB in confounding or measurement of outcome domains; - Among the 17 RCTs, 13 studies had a high RoB mostly due to a deviation from intended intervention, missing outcome data, and a bias in the measurement of the outcome, other 4 studies were observed to have some concerns in potential bias related to deviations from intended interventions, randomization process, and measurement of outcomes |
| Goodman et al. 2021 [24] | Systematic Review | UK | The risk of bias for RCTs and nonrandomized studies was assessed using the Mixed Methods Appraisal Tool. | - The RCTs were of a broadly high quality, 6 studies did not have complete outcome data at follow-up; - The nonrandomized trials studies quality were again high, apart from the included studies that did not control for confounders in their analysis. |
| Finucane et al. 2021 [25] | Systematic meta-review | UK | The AMSTAR 2 critical appraisal tool was applied for systematic reviews. | Only one review was judged as moderate quality, overall quality of all other reviews was rated low (n = 15) or very low (n=5). |
| Li et al. 2021 [14] | Systematic review and meta analysis | China | Included studies quality assessment were used the Cochrane Handbook for Systematic Reviews of Interventions Version 5.1.0 RCT. | The quality of the included studies was assessed as "B". |
| Naoum et al. 2021 [13] | Systematic Review | Greece | All studies included in the final analysis were evaluated for their methodological quality using the Drummond Checklist for assessing economic evaluations. | The methodological quality of the included studies was good. |
| Cameron and Munyan 2021 [30] | Systematic Review | USA | The Methodological Rigor Scoring Instrument (MRSI) was used to assess the quality. | - 6 quantitative methodological studies had a mean score of 12.1 (range of 7–15) representing low to medium strength of evidence. - 7 qualitative methodological studies scored ranged from 5 to 11 of a possible total score of 15, indicating low to medium strength of evidence. |
| Archer et al. 2021 [26] | Systematic Review | UK | The quality of the included studies was assessed by the lead author using the Hawker Checklist. | Papers demonstrated fair or good quality reporting. |
| Bienfait et al. 2020 [41] | Systematic Review | France | Not reported | Not reported |
| Hancock et al. 2019 [27] | Systematic Review | UK | Using guidance provided by Wallace et al’s 2004 paper, the authors appraised the methodology of 19 of the 30 papers. For the 11 which were not able to be assessed, this was due to the paper being descriptive in nature with insufficient detail on study design. | 8 of the 19 papers met all of the nine criteria completely or to some extent; the maximum score was 18. |
| Jess et al. 2019 [42] | Systematic Review | Denmark | The quality of the included studies was used a tool developed by Hawker et al. | The studies received between 20 and 36 points with an average score of (27.4±4.6). |
| Allsop et al. 2018 [28] | Systematic Review | UK | Not reported | Not reported |
| Bush et al. 2018 [31] | Systematic Review | USA | Not reported | Not reported |
| Head et al. 2017 [32] | Systematic Review | USA | The Cochrane Collaboration’s tool for assessing risk of bias in randomized trials was used to evaluate study rigor and quality of the articles with quantitative results;For studies reporting qualitative results, a checklist was used to evaluate whether articles included methodologies used to ensure adequate trustworthiness. | - Of the 6 quantitative studies, 3 scored as having moderate quality/rigor, whereas the remaining 3 scored as low quality; - Of the 6 studies reporting qualitative results reported 5 different methods for ensuring trustworthiness, whereas 1 article reported 4, 1 reported 3, and 1 article reported 2 methods |
| Zheng et al. 2016 [33] | Systematic Review | USA | The Cochrane Collaboration’s tool for assessing risk of bias in randomized trials was used to evaluate study rigor and quality. | Of the 9 studies, the majority (77.8%) scored as moderate. |
| Ostherr et al. 2016 [34] | Systematic Review | USA | Procedures for coding included methods for assessing risk of bias, based on the Cochrane Collaboration’s recommendation in support of using a domain-based evaluation. | Of the 38 studies, the majority scored as low. |
| Capurro et al. 2014 [40] | Systematic Review | Chile | Not reported | Not reported |
| Bradford et al. 2013 [38] | Systematic Review | Australia | Each article included in the review was evaluated for validity using the appropriate grading tool for its study design, from a suite of tools designed by the Critical Appraisal Skills Programme (CASP). | Of the 33 studies, the majority scored as high. |
| Oliver et al. 2012 [35] | Systematic Review | USA | Articles are scored with a methodology score from EITHER Part IA (Quantitative article) or Part IB (Qualitative scoring) AND a pertinence score using Part II(Scoring criteria adapted from Higginson). | - The mean score for quantitative studies was 9.2 (range of 5-14) representing low-medium strength evidence; - The mean score of the qualitative evidence was 9 out of 11 (range of 5-11), representing medium-high strength of evidence; - The total pertinence score averaged 12 (range of 10-14) out of a possible 15 for both quanti_x005ftative and qualitative articles. |
