# Supplementary material for: Role of Digital Health on Palliative Care: Umbrella Review
Source: J Med Internet Res. 2025 Oct 28;27:e72104. doi: 10.2196/72104 (PMC12605284; doi:10.2196/72104)
Supplement: Multimedia Appendix 5 [file jmir_v27i1e72104_app5.docx]

| Authors and year published | Q1* | Q2 | Q3 | Q4 | Q5 | Q6 | Q7 | Q8 | Q9 | Q10 | Q11 |
| --- | --- | --- | --- | --- | --- | --- | --- | --- | --- | --- | --- |
| Yang et al. 2024 [15] | Y | Y | Y | Y | Y | Y | Y | Y | N | Y | Y |
| Dilhani et al. 2024 [22] | Y | Y | Y | Y | Y | Y | Y | Y | N | U | Y |
| Johansson et al. 2024 [23] | Y | Y | Y | Y | Y | U | Y | Y | N | Y | U |
| Chen et al. 2023 [36] | Y | Y | Y | Y | Y | Y | Y | Y | N | Y | Y |
| Xu et al. 2023 [37] | Y | Y | Y | Y | Y | Y | Y | Y | N | Y | Y |
| Sánchez-Cárdenas et al. 2023 [43] | Y | Y | Y | Y | Y | U | U | Y | N | U | Y |
| Steindal et al. 2023 [39] | Y | Y | Y | Y | Y | Y | Y | Y | N | Y | Y |
| Kamalumpundi et al. 2022 [29] | Y | Y | Y | Y | Y | U | Y | Y | Y | Y | Y |
| Goodman et al. 2021 [24] | Y | Y | Y | Y | Y | Y | Y | Y | N | U | Y |
| Finucane et al. 2021 [25] | Y | Y | Y | Y | Y | Y | Y | Y | N | Y | Y |
| Li et al. 2021 [14] | Y | Y | Y | Y | Y | Y | Y | Y | N | Y | Y |
| Naoum et al. 2021 [13] | Y | Y | Y | Y | Y | U | Y | Y | N | Y | U |
| Cameron and Munyan 2021 [30] | Y | Y | Y | Y | Y | Y | Y | Y | N | Y | Y |
| Archer et al. 2021 [26] | Y | Y | Y | Y | Y | Y | Y | Y | N | Y | Y |
| Bienfait et al. 2020 [41] | Y | Y | Y | Y | NA | NA | U | U | N | U | Y |
| Hancock et al. 2019 [27] | Y | Y | Y | Y | Y | Y | Y | Y | N | Y | Y |
| Jess et al. 2019 [42] | Y | Y | Y | Y | Y | Y | Y | Y | N | Y | Y |
| Allsop et al. 2018 [28] | Y | Y | Y | Y | NA | NA | Y | Y | N | Y | Y |
| Bush et al. 2018 [31] | Y | Y | Y | Y | NA | NA | Y | Y | N | Y | Y |
| Head et al. 2017 [32] | Y | Y | Y | Y | Y | Y | U | Y | N | Y | Y |
| Zheng et al. 2016 [33] | Y | Y | Y | Y | Y | Y | U | Y | N | Y | Y |
| Ostherr et al. 2016 [34] | Y | Y | Y | Y | Y | U | Y | Y | N | Y | Y |
| Capurro et al. 2014 [40] | Y | Y | Y | Y | NA | NA | Y | Y | N | Y | Y |
| Bradford et al. 2013 [38] | Y | Y | Y | Y | Y | U | U | Y | N | Y | Y |
| Oliver et al. 2012 [35] | Y | Y | Y | Y | Y | U | Y | Y | N | Y | U |

Source: Joanna Briggs Institute (2017) Checklist for Systematic Reviews and Research Syntheses N=No; NA=Not Applicable; U=Unclear; Y=Yes.

*Items by question number

1. Is the review question clearly and explicitly stated?.

2. Were the inclusion criteria appropriate for the review question?.

3. Was the search strategy appropriate?.

4. Were the sources and resources used to search for the studies adequate?.

5. Were the criteria for appraising studies appropriate?.

6. Was the critical appraisal conducted by two or more reviewers independently?.

7. Were there methods to minimize errors in data extraction?.

8. Were the methods used to combine studies appropriate?.

9. Was the likelihood of publication bias assessed?.

10. Were recommendations for policy and practice supported by the reported data?.

11. Were the specific directives for new research appropriate?.
